# Minimal Residual Disease in Multiple Myeloma: Current Landscape and Future Applications With Immunotherapeutic Approaches

**DOI:** 10.3389/fonc.2020.00860

**Published:** 2020-05-27

**Authors:** Ioannis V. Kostopoulos, Ioannis Ntanasis-Stathopoulos, Maria Gavriatopoulou, Ourania E. Tsitsilonis, Evangelos Terpos

**Affiliations:** ^1^Department of Biology, School of Science, National and Kapodistrian University of Athens, Athens, Greece; ^2^Department of Clinical Therapeutics, School of Medicine, National and Kapodistrian University of Athens, Athens, Greece

**Keywords:** multiple myeloma, minimal residual disease, prognostic factor, primary endpoint, therapeutic intervention

## Abstract

The basic principle that deeper therapeutic responses lead to better clinical outcomes in cancer has emerged technologies capable of detecting rare residual tumor cells. The need for ultra-sensitive approaches for minimal residual disease (MRD) detection is particularly evident in Multiple Myeloma (MM), where patients will ultimately relapse despite the achievement of complete remission, which is commonplace due to remarkable therapeutic advances. Consequently, current response criteria on MM have been amended based on MRD status and MRD negativity is now considered the most dominant prognostic factor and the most valuable indicator for a subsequent relapse. However, there are particular limitations and several aspects for MRD assessment that remain open. This review summarizes current data on MRD in the clinical management of MM, highlights open issues and discusses the challenges and the endless opportunities arising for both patients and clinicians. Furthermore, it focuses on the current status of MRD in clinical trials, its dynamics in addressing debatable aspects in the clinical handling and its potential role as the prevailing factor for future MRD-driven tailored therapies.

## Introduction

The extended research and coordinated efforts to understand the biology and the clinical aspects of Multiple Myeloma (MM) has currently led to the development of novel regimens, drugs, and therapeutic approaches which offer a clear benefit in favor of the patients. The therapeutic efficacy is reflected by the massive increase of the number of patients achieving complete remission (CR), followed by extended periods free of progression. Nevertheless, MM still remains an incurable disease with relapses that would eventually lead to uncontrollable disease and death.

Based on the basic principle that the deeper the remission the better controllable the disease, it is of utmost clinical significance to be able to assess the efficiency-depth of a selected treatment and thus anticipate an eventual relapse. The presence of Minimal Residual Disease (MRD), i.e., minute numbers of myeloma cells that may remain in the bone marrow (BM) of the patient after treatment, has been proved crucial for monitoring remission status and is regarded as the major cause of relapse. Current technology allows for the detection of MRD at levels as low as one myeloma cell in one million of total examined cells, thus providing brand new opportunities for both clinicians and patients.

## State of the Art Methods for MRD Assessment

The significance of MRD in the clinical setting of MM has long been reported (1–4), though its clear effect has been widely appreciated with the development of more sensitive techniques. Traditional molecular methods, i.e., allele-specific oligonucleotide PCR (ASO PCR) or real-time quantitative PCR (ASO RQ-PCR) (5–7) has been replaced by next-generation sequencing (NGS), while the 4, 6, or 8-color multicolor flow cytometric (MFC) approaches have been replaced by Next-Generation flow cytometry (NGF) (8) or other similar multicolor panels of high sensitivity (9). ASO RQ-PCR is a widely used and affordable technique using ASO primers coupled with fluorescent probes for the real-time amplification and detection of the clonal rearrangement of the immunoglobulin heavy chain variable region (VDJ-IgH). However, the need for patient-specific primers along with technical issues due to high level of IgH somatic hypermutation constitute the major weaknesses of this approach, that can be applicable only for 60–70% of the cases (10, 11).

Based on current International Myeloma Working Group (IMWG) response criteria (12), the presence of MRD in CR patients should be tested with either NGF or NGS (or a validated equivalent technique) with a minimum sensitivity level of 10^−5^. It is generally implied that the MRD detection power is superimposed by the utilization of either of the two techniques, the preference of which lays on local availability. However, each approach has specific pros and cons (Table 1).

**Table 1:** Technical features of NGF and NGS for MRD detection.

	**NGF**	**NGS**
Applicability (% cases)	99%	~90%
Sensitivity	2–4 × 10^−6^	10^−6^
Time to result	2–3 h	≥7days
Number of cells required	2 × 10^7^	2–3 × 10^6^
Need for fresh sample	Yes (within 24 h)	No
Need for diagnostic sample	No	Yes
Quantitative	Yes	Yes
Intrinsic quality control for hemodilution	Yes	No
Cell characterization	Yes	No
Molecular characterization	No	Yes
Availability	Wide	Limited
Reproducibility among centers	High	Not reported
Harmonization	Yes	Not reported
Cost	+	++

The sensitivity of NGS is higher than that of NGF and can be used for detection of rare residual myeloma BM cells at the level of 10^−6^. There are not many reports comparing the frequency of MRD positivity when using both techniques. Recently, the results of the FORTE trial for patients achieving very good partial response or better (≥VGPR) pre-maintenance has compared the MRD data analyzed both by NGS and second (2nd)-generation MFC (sensitivity 10^−5^) and revealed discordances in 54/176 (30%) of analyzed samples. In all but one of these discordances, MRD positivity was missed by MFC. However, when NGF -instead of 2nd generation MFC- (sensitivity 10^−5^- 10^−6^) was compared with NGS in a subgroup of patients of the same study, results were highly concordant with the two techniques (13). Another advantage of NGS is that it can be applied retrospectively on stored material including not only cryopreserved cells but also archival BM slides (14). On the other hand, NGS requires a diagnostic ID sample for the detection of the patient-specific clonal re-arrangement and has a slightly lower applicability than NGF (ca. 90 vs. 99%), as in some patients the dominant clonal sequence of myeloma cells in ID samples cannot be detected, mainly due to high-level somatic lg hypermutation that affect the primer-binding sites (15, 16). Moreover, though more sensitive, the bioinformatic analysis of sequencing data for MRD positive cases with very low tumor burden is complicated and requires enough quantity of input DNA to be deeply sequenced to discriminate the minute clonal sequence from experimental background. The most commonly utilized NGS-based ClonoSEQ (Adaptive Biotechnologies) platform for MRD evaluation has overcome many of the technical pitfalls seen in NGS technology; however the cost is high and requires specialized centers for sample preparation and data interpretation, which, in its current form, makes it problematic for daily clinical management (17).

The major advantage of NGF is its high applicability in 99% of MM patients and the relatively simple manual set-up in diagnostic labs equipped with the appropriate 8-color cytometers, following the standardized EuroFlow guidelines. The cost of the technique is significantly more affordable and the results may be available within a few hours upon BM aspiration. There is no need for a prior diagnostic sample evaluation due to the elegantly elaborated 8-color marker combinations that can efficiently discriminate between normal and aberrant plasma cells in the whole spectrum of intra-phenotypic heterogeneity (8, 18, 19). Furthermore, NGF methodology allows for an intra-quality control check for hemodiluted samples—the major pitfall for both NGF and NGS approaches- by identifying cellular components (i.e., mast cells, B cell precursors, erythroblasts) that are mainly present in the BM. This point is commonly underestimated, though it consists one of the major advantages of NGF; the multiparametric panels allow for the global characterization of BM cells, providing valuable information for the tumor microenvironment and the individualized immune profiling of the patient at the time of MRD examination. Obviously, the applied panel for MRD detection has not been selected on the basis of a thorough BM cell characterization; however it can indirectly provide quantitative information regarding the relative abundance of distinct BM cell populations, as well as the differential count of the various subsets constituting a main cell population. A main disadvantage of NGF is the requirement of a fresh sample that should be processed within 24 h post aspiration, and the need for a relatively higher amount of cells compared to the cell numbers needed for NGS assays. Moreover, contrarily to NGS, NGF is unable to provide molecular information on the MRD cells, in terms of detecting clonal hierarchical changes and clonal evolutionary events that are common during the natural history of MM (20–22).

## Alternative Approaches for MRD Detection

Apart from the methodological restrictions, the particular nature of MM may lead to specific pitfalls that need to be considered. Different areas of the BM may have various infiltration rates due to the patchy pattern of the disease. Most importantly though, distinct clonal subsets may home to different areas of the BM reflecting a spaciously molecular heterogeneity (23), thus questioning the representativeness of information obtained by sampling a very specific area. Additionally, the presence of extramedullary disease (EMD) may lead to false-negative MRD results, since these myeloma cells are not sampled for the standard BM MRD assays. This is particularly challenging for patients at later stages of the disease where EMD is more commonly developed (24, 25). These limitations, together with patients' discomfort for BM invasive methods have led to efforts for alternative approaches to overcome these issues.

## Imaging Methods

Different imaging techniques have been employed to evaluate response to treatment, which independently or concomitantly with BM MRD assays may discriminate patients with higher risk for relapse. The utilization of ^18^fluoro-2-deoxyglucose positron emission tomography (FDG-PET)/computed tomography (CT), able to distinguish lesions with metabolically active disease, may prove really useful for patients with EMD, and has already been included as a separate subcategory in the latest IMWG response criteria (12). The absence of metabolically active areas after treatment has long been correlated with improved clinical outcomes, frequently as an independent predictor (26, 27). Of note, the advantageous impact of PET-negative results has been reported on various phases of administered therapy (before or after transplantation, before maintenance therapy etc.) (28, 29). For example, Nanni et al. (28) examined the impact of achieving PET-CT negativity after ASCT for MM patients found PET-positive at diagnosis. PET-negativity (and therefore PET-CR) was considered when areas of increased tracer uptake found at baseline had disappeared, and PET improvement (PET-PR) was considered when the number of sites of FDG uptake had decreased and/or the maximum standardized uptake value (SUVmax) of the lesions had declined more than 20%. Their results highlighted that the achievement of PET-CR after treatment was correlated with longer progression free survival (PFS: 27, 6 months for PET-negative vs. 18 months for PET positive, *P* < 0.05) whereas the SUVmax ≥4,2 after treatment was an independent unfavorable prognostic factor. Similarly, data from the IMAJEM study (29) showed that the PET-CT normalization before maintenance therapy for MM patients found positive at baseline resulted in improved clinical outcomes both in terms of PFS (30-month PFS: 72% for normalized PET-CT vs. 56,8% for those remained PET-CT positive, *P* = 0.011) and overall survival (2-year OS rate: 94,7% for normalized PET-CT vs. 72.9% for those who remained PET-CT positive, *P* = 0.033).

Magnetic resonance imaging (MRI) is an alternative sensitive approach for detecting diffuse focal lesions and recent data have highlighted its promising role for evaluating to treatment. The results from the IFM/DFCI 2009 trial showed that there are no major differences between PET-CT and MRI in their ability to detect bone lesions at diagnosis, though there were 17/134 (12.7%) discrepancies between the two methods (29). Nevertheless, FDG-PET/CT remains the preferred imaging approach for monitoring EMD response, though improved and more sensitive MRI approaches [i.e., diffusion-weighted imaging (DWI) or dynamic contrast-enhanced (DCE) MRIs] are likely to superior for evaluating response efficacy after treatment (29–32).

At present, in their current form, imaging technologies have some limitations and at certain conditions may lead to both false positive and false negative results. Recent data have depicted that PET findings could not associate strongly with clinical responses in the context of MRD detection (33); obviously the current lack of standardization and uniform criteria for imaging parameters and interpretation is limiting the potential of imaging as a dominant methodology in MRD evaluation (34). However, as imaging techniques are becoming more sensitive and informative, their actual role in MRD assessment is likely to increase extensively in the near future, at least as surrogate to BM-assays. In this context, ImmunoPET, a new functional imaging approach that uses radiolabeled monoclonal antibodies against targeted antigens, may prove a really valuable and highly specific tool for tracking disease out of the BM, as imaging will be applied to tumor cells based on antigen expression, independently of metabolic processes. The development and standardization of such techniques would be of utmost significance in the era of novel agents and targeted immunotherapies.

## Liquid Biopsy

Liquid biopsy has been proposed as an alternative approach for tracking residual disease after treatment. An effective and sensitive peripheral blood (PB) testing that would be able to mirror BM and/or EMD status, monitor disease kinetics and predict a subsequent relapse would theoretically be the ideal assay, attractive for both clinicians and patients. Current approaches for liquid biopsy testing have mainly focused on three elements; circulating tumor DNA (ctDNA), circulating tumor cells (CTCs) and serum monoclonal immunoglobulins.

CtDNA comprises degraded DNA fragments released in the bloodstream from cancer cells and constitutes a molecularly distinct DNA fragment of the total cell-free DNA (cfDNA). Elevated levels of ctDNA have been detected in cancer, especially in advanced stages; hence ctDNA has emerged as a promising and valuable biomarker for neoplasias and solid tumors. Using an ultra-deep sequencing approach targeting all protein-coding exons of a 5-gene panel for paired ctDNA and BM samples, Kis et al. (35) have recently reported a 96% concordance in detecting tumor-derived mutations with allele fractions (AF) as low as 0.25% between ctDNA and BM paired samples, with a specificity value of >98%. Most importantly, the study provides evidence for an effective reconstruction of subclonal hierarchies through the analysis of blood plasma that may be even more detailed than the one derived by single BM aspirates, since the authors reported 3 cases with detectable AFs only in the ctDNA but not in their matched BM samples.

On the contrary, the utility of ctDNA for monitoring MRD is still ambiguous. Pilot studies with amplicon-based NGS approaches for IgH gene re-arrangement in the ctDNA of diagnostic and post-treatment samples have resulted in controversial results. Biancon et al. (36) reported that ctDNA analysis was clinically relevant and could be used for the evaluation of MRD during therapy in CR patients, since IgH ctDNA levels were significantly correlated with MRD levels obtained by 8-color flow cytometry, as well as with other parameters such as BM plasma cells on trephine biopsies, M-protein concentrations and sFLC ratio. Contrarily, Oberle et al. (37) reported that the apparently detectable ctDNA of diagnostic samples, could be tracked only in 39% of patients with VGPR or worse response, similarly to the study of Mazzoti et al. (38) where ctDNA was undetectable in 69% of samples which have been found clearly MRD positive in the BM. Additionally, in a recent study employing tumor-specific ASO-qPCR for ctDNA detection, correlation between ctDNA after treatment and flow-MRD status was only partially corresponding (39). It should be noted though that ctDNA may decline more rapidly in effective treatments than other plasma compartments as well as PB and BM residual disease clearance may follow different dynamics (37, 40, 41).

Overall, current evidence show that ctDNA may be used for mirroring the genetic landscape of the BM-based disease and recapitulate efficiently the spatial intra-subclonal heterogeneity. At present, the employment of ctDNA as a tool for MRD monitoring is not totally feasible and requires systematic efforts toward integration of molecular techniques, combined with the appropriate bioinformatic tools to form a sensitive and unified platform that will expand the targeted genomic regions and eliminate the background of the non-tumor cell-free compartment. However, the evaluation of ctDNA may be used complementary to BM testing providing valuable clinical and biological information of the disease status, especially in later disease stages and/or in cases with an extramedullary involvement.

CTCs constitute the second component of liquid biopsy with clinical utility. The increased frequency of CTCs has been proposed as an unfavorable prognostic factor for MM patients both at diagnosis, after autologous stem cell transplantation (ASCT) or during relapse (42–45). Different approaches with various sensitivities have been used for the enumeration of CTCs including immunofluorescence, MFC of 2, 4, 6, or 8-colors and molecular approaches. Phenotypic and molecular characterization of CTCs has proven the feasibility of CTCs to recapitulate the disease of the BM, despite the fact that they appear some unique phenotypic and functional features (46, 47). The comprehensive genomic characterization of CTCs via targeted (48) and non-targeted (47, 49) whole-exome sequencing has revealed a high concordance in clonal mutations between CTCs and BM paired samples with some subclonal mutations being exclusively found in CTCs. Similarly to ctDNA, CTCs may depict a more representative portrayal of the disease than BM samples obtained from one area, especially for patients with EMD.

The NGF assay for MRD evaluation is also feasible and highly sensitive for the detection and enumeration of CTCs in MM (50–52). In a different approach, an automated technique (CELLSEARCH, Menarini Silicon Biosystems) based on the differential expression of CD19, CD45, and CD38 has been recently developed for isolation and enumeration of CTCs out of total PB cells (53). Both techniques have been applied in the clinical setting and highlighted the potential of CTCs to be used as a useful prognostic tool, complementary to the established methods. Nevertheless, the applicability of these techniques for MRD evaluation based only on CTCs is currently unachievable and much standardization needs to be performed to contrast the BM-based assays. As sequencing costs are expected to decrease in the near future, more sensitive molecular techniques aiming at small input material may be more feasible for a non-invasive future PB-based MRD assay (17, 54).

MM is characterized by the presence of monoclonal immunoglobulin (M-protein) in the serum and urine of patients, so that serum M-protein could be used as an alternative biomarker for MRD tracking. Though the classical electrophoresis is not suitable for the detection of minute M-protein levels, the development of new highly-sensitive mass spectrometry assays could potentially serve to this purpose.

The clonotypic peptide method recognizes unique patient-specific peptide(s) of the M-protein complementarity determining region (CDR) which can be followed over time (55). This approach is 2000 times more sensitive than conventional electrophoretic assays, but it requires diagnostic sample, advanced bioinformatic tools to identify patient-specific unique peptides, is costly with long turn-around time and may not be applicable to all patients. Another mass spectrometry method is the monoclonal immunoglobulin rapid accurate molecular mass (miRAMM), which takes into account the accurate mass of the clonal light chain as a tracking parameter (56). This method is quicker and has a simpler analysis process than the clonotypic peptide assay. Furthermore, miRAMM is able to distinguish among M-protein spikes and other monoclonal antibody drugs, which are widely used in current clinical setting, it allows for the detection of several co-existing subclones in a patient's sample—hence enabling the monitoring of clonal evolutionary events over time—and finally can discriminate several post-translational 0modifications (57). On the other hand, miRAMM still needs a diagnostic sample, is less sensitive than the clonotypic peptide assay and also requires special equipment, which is unavailable for the majority of the diagnostic labs. Both techniques had been already used for MRD detection in MM with promising results (58, 59). However, these assays are relatively new with several open issues to be addressed, thus not constituting the current preferable choice for MRD evaluation. Future improvements and additional studies may show if these assays could be adapted for more routine use in daily clinical practice (57).

## MRD as a Prognostic Factor

There is no doubt that the achievement of MRD negativity confers a more favorable outcome for treated MM patients. The first meta-analysis by Landgren et al. (60) and the one that followed by Munshi et al. (61) including more studies with older therapies and various approaches for MRD detection have verified the prognostic impact of MRD negativity in the clinical outcome. The latter meta-analysis showed a 59% reduced risk of progression and 43% reduced risk of death for MRD negative patients with a median PFS of 54 vs. 26 months and a median OS of 98 vs. 82 months for MRD negative vs. MRD positive patients, respectively.

When compared with other prognostic factors, MRD has been shown to be superior and the most relevant predictor of clinical outcome. In multivariate analyses, the achievement of MRD negativity is proven to be the strongest independent prognostic factor which surpasses other favorable prognostic parameters (60–65). The first point to be particularly emphasized is that the clinical impact of MRD negativity is also apparent in patients' subgroups with ominous prognostic features. Current data support that the risk is dynamic and adverse prognostic features may be reversed upon achieving MRD negativity (64, 66, 67). Recently, Paiva et al. (66) reported that patients' risk at diagnosis estimated by R-ISS disease stage can be modulated upon achieving deep responses by MRD negativity and the initial R-ISS prognostic stratification is meaningful only in patients with persistent MRD. These important findings underscore the weakness of current prognostic criteria to fully and efficiently predict patients' outcome due to the apparent disease heterogeneity, whereas highlight the R-ISS importance in predicting early vs. late disease progression for those with persistent MRD. Similarly, patients with high-risk baseline cytogenetics who achieved MRD negativity after treatment, had significantly improved outcomes when compared with MRD persistent counterpart, but most importantly, they experienced similar survival outcomes with standard-risk patients who also achieved MRD negativity (64, 67, 68). However, it should be noted that this may not be the case for the whole spectrum of high-risk aberrations. In a study addressing the impact of MRD after ASCT in high-risk cytogenetics, Chakraborty et al. (69) reported that MRD negativity can overcome the unfavorable prognosis of *t*_(4;14)_ but not of del(17p13) or the concomitant presence of more than one adverse aberrations. It is also noteworthy that only 3/28 (11%) of patients with del(17p) did manage to achieve MRD negativity in the IFM 2009 clinical trial (67).

The second point that should be highlighted is that the favorable prognostication of MRD negativity stands independent of the assigned treatment. This has been shown in various aspects of the therapeutic management when MRD was evaluated after induction, ASCT, consolidation or during various phases of maintenance therapy and for different patient categories including eligible and non-eligible for transplantation as well as patients in the relapsed/refractory setting (66, 67, 70–78). In major clinical trials comparing different therapeutic approaches or different combination regimens as part of the assigned treatment of any phase, the beneficial value of MRD negativity remained similar irrespective of the prior therapeutic approach, thus forming a new grouping of MRD positive and MRD negative patients, distinguished only by the different risk for further disease progression (79).

## CR, 10^−5^, 10^−6^ or Even Deeper?

Current therapeutic improvements have substantially increased the frequency of patients achieving CR (80), thus necessitating the evaluation of a superior prognostic factor for therapeutic response. The independent prognostic value of MRD among all clinical parameters evaluated in the recent meta-analyses was impressively verified in the subset of patients achieving CR after treatment, thus signifying that MRD negativity is a superior prognostic factor than conventional CR. More recently, the results from the 3 PETHEMA/GEM clinical trials have clearly highlighted this divergence, demonstrating that patients in CR that are MRD positive post treatment have no better outcomes than patients in partial response (PR) (64). The superiority of MRD toward CR sensibly indicates that all patients in VGPR or less would be MRD positive but this is not always the case (81). Tumor elimination and clearance of serum upon an effective treatment may follow different dynamics, as the half-life of IgA and IgG is relatively high (~3–4 weeks) (82), and thus the detection of M-protein may not necessarily reflect the persistence of clonal cells in the BM. This is an important aspect of the disease, especially in the era of novel compounds and highly effective immunotherapies, where tumor elimination may be really fast (83). However, even in these cases, VGPR is about to turn to CR soon, thus suggesting that MRD evaluation should be tested in the context of CR (12, 66), given that the early achievement of MRD negativity does not bring any clear advantage compared to MRD negativity achieved at a later point (66, 67).

Apparently, this is a new era where clinicians are moving away from the traditional response criteria and MRD assessment has become the new gold standard for treatment efficacy. Nonetheless, what is the optimal threshold that MRD should be tested? And even if we set a specific threshold, should the evaluation of MRD focus only on the positive/negative result or should we also pay attention to the quantitative parameters of an MRD positive result? As for the latter question, there are several studies depicting the quantitative effect of the number of residual clonal cells on a logarithmic scale, defining distinct groups of patients with different outcomes (62, 67, 84, 85). Regarding the ideal threshold, the limit for MRD detection is set to 10^−5^ based on current response criteria (12), though there is now evidence that a more sensitive set-up limit of 10^−6^ is clinically relevant. Perrot et al. (67) using NGS and Paiva et al. (66) utilizing NGF showed that patients who achieve MRD negativity at the level of 10^−6^ have prolonged progression-free periods when compared to those who are MRD negative at 10^−5^ or higher. In other words, this means that the 10^−5^ MRD negative group is heterogeneous and contains also MRD positive patients with rare residual cells that may be falsely missed due to sensitivity restrictions.

Following this paradigm, it seems that the deeper the response the better the outcome for MM patients, so an ultra-sensitive assay of 10^−7^ limit may prove clinically informative in the near future. At present though, 10^−6^ seems to be the most optimal threshold for MRD detection, and current criteria are likely to be reconsidered based on this new evidence. In the same context, it is rationale that an updated version of response criteria would take advantage of the quantitative nature of the sensitive MRD assays and may stratify patients not only on the context of MRD positive and MRD negative but on the level of MRD positivity as well (86). This stratification would be helpful for deeper evaluation of treatment efficacy and may prove important for defining different therapeutic MRD-driven strategies based on the level of response to a previous regimen.

## Limitations and Improvements for Undetectable MRD

Despite the clear clinical impact of achieving MRD negativity at the level of 10^−6^, biological relapses may still occur at a significant rate (33, 66, 67, 84) Perrot et al. (67) reported that 29% of patients with MRD <10^−6^ by NGS experienced disease progression after a median follow-up of 55, 50, and 38 months from randomization, start, and completion of maintenance therapy, respectively. Using the NGF approach Paiva et al. (66) showed that 14/208 (7%) of patients with undetectable MRD at 10^−6^ level had relapsed after a median follow-up period of 40 months post consolidation therapy. Interestingly, 6/14 (43%) cases showed extraosseous plasmacytomas at relapse -either at a pre-existing EMD background (4 cases) or as *de novo* presentation of new plasmacytomas (2 cases)- without detectable M-protein or BM infiltration. Moreover, the recent results from the CASSIOPET study, the PET/CT companion study of CASSIOPEIA trial, have depicted a concordance of 61.9% between MRD negativity and PET-CR post consolidation. In particular, 102/176 cases were concordant, whereas there were 12 patients (6.8% of all cases) with PET-CT positivity and absence of MRD clonal cells (87). These observations demonstrate the failure of BM-based assays to detect MRD in these cases, on the basis on a false-negative interpretation. On the other hand, they necessitate the combined application of MRD assays with functional imaging to monitor treatment response, especially in those patients presented with macrofocal or EMD (23, 29, 87, 88).

However, not all missed MRD positive cases could be justified by an extramedullary involvement. There may be cases where MRD is present at levels lower than the achieved limit of detection (LOD). Nevertheless, as the current methods for MRD assessment do not allow for a practical and cost-effective adaptation for reaching levels lower than 10^−6^, we may need to look for additional prognostic parameters prior or after the administered therapy that will complement MRD testing. For example, Lahuerta et al. (64) reported that the presence of a MGUS-like profile at diagnosis (i.e., the presence of >5% of normal plasma cells in total plasma cell compartment) could further discriminate among MRD negative patients, those with a minimum risk of progression, since MGUS-like MRD negative patients had a median PFS of 148 vs. 61 months and 44 months for MM-like MRD negative and MGUS-like MRD positive subgroups respectively.

The evaluation of individualized immune profiling upon a treatment intervention could also serve to this direction, adding prognostic value to the MRD assay (89–92). Paiva et al. (89) have shown that distinct immune profiles composing by different levels of erythroblasts, B cell precursors and memory B cells correlate with different clinical outcomes, and this impact was independent by the MRD status coefficient. Accordingly, we have recently shown that the turnover of MRD negativity to MRD positive status in sequential MRD monitoring is accompanied with specific changes of immune composites within the BM including a relative increase of erythroblasts, NK cells and tumor-associated macrophages (90). In another interesting study, Botta et al. (92) have highlighted the prognostic impact of the ratio between the CD27-/CD27+ T cells in the BM microenvironment providing also evidence that the CD27- T cell compartment is mainly consisted by clonotypic effector/exhausted CD8+ reactive T cells.

These data point out the clinical relevance of immune profiling in MM and highlight the dynamics of immune monitoring during the course of the disease. The BM niche may support the clonal cells but it also hosts several immune subsets trying to control tumor growth. Alterations in the interaction between myeloma cells and their microenvironmental components may lead to immune escape mechanisms with subsequent relapses and uncontrollable disease (93–95). In this context, the absence of MRD at a specific examination point does not ensure a permanent MRD negativity. MRD reappearance may occur at any point and this recurrence can predict subsequent responses (90, 96, 97). The optimal time point for sequential MRD monitoring is yet to be standardized; a 12-month time-interval between two consecutive evaluations is often preferred, though in the relapsed/refractory setting a shorter interval would be more sensible. Nevertheless, the sustained 2-year period of MRD negativity has been reported to identify patients with a very low risk of disease progression (97).

## MRD as an Endpoint in Clinical Trials

MRD evaluation has been widely accepted as a robust method for treatment efficacy and the MRD negativity rate is widely implemented as an endpoint in contemporary clinical trials (98, 99). However, until recently the vast majority of clinical trials have not included MRD as a primary clinical trial endpoint. As discussed throughout the present text, several aspects of MRD assessment are still open and have not been optimized including: (i) patient selection (those in sCR and CR or VGPR as well?), (ii) optimal time points of MRD assessment during the treatment course, (iii) optimal cut-offs (10^−5^ or 10^−6^?), (iv) frequency of MRD monitoring following a MRD negative result, (v) likelihood and interpretation of false positive and false negative results, (vi) evaluation of MRD only in the BM or in PB as well, (vii) necessity of multimodal MRD assessment with different techniques and imaging approaches, (viii) evaluation of the quantitative tumor burden and further stratification for MRD positive patients and ix) evaluation of additional prognostic features that may complement the MRD result. In this context, MRD may be considered as an intermediate endpoint from the regulatory authorities.

Both the FDA and EMA (guideline EMA/CHMP/459559/2018) are eager to consider a novel drug or combinations that comply an unmet medical need for patients with MM, for accelerated approval based on MRD data. However, such a conditional approval should be followed by mature data on robust and well-established clinical trial endpoints, such as OS and PFS, supporting the initial MRD results. Recently, a large meta-analysis encompassing data from 6 randomized clinical trials (3283 newly diagnosed MM patients) showed that the achievement of MRD negativity was strongly correlated with prolonged PFS (79). The coefficient of determination for the weighted regression line was 0.97, and therefore MRD met the Prentice criteria (100) for PFS surrogacy.

There is no doubt that the standard PFS and OS endpoints may provide the most unambiguous evidence for the efficacy of a tested drug, combination therapy or a new treatment strategy, compositing measures of response rate, depth of response, toxicity, intolerance or long-term benefit of a particular regimen. However, current improvements in MM management have substantially prolonged the median value of progression free periods and survival, even in the setting of relapsed/refractory disease. This prolongation makes prospective clinical trials lasting and costly, thus entailing the need for early markers of efficacy that can reliably mirror the longer term outcomes. MRD could serve these prerequisites and currently many ongoing or planed trials have set MRD as an exclusive or additional primary endpoint to PFS and/or OS (Table 2).

**Table 2:** Ongoing clinical trials including MRD as a primary clinical endpoint.

**Identifier**	**Phase**	**Regimen**	**Subjects**	**Primary outcome measures**	**Sensitivity/method**	**Status**
NCT04288765	3	DaraKRd ± ASCT	56 NDMM eligible or not for ASCT	MRD negativity rate	NR/MFC	Recruiting
NCT04287855	2	Single arm: Isa-KPd	90 R/R MM	MRD negativity rate	10^−5^/NR	Not yet recruiting
NCT04268498	2	DaraKRd vs. KRd vs. VRd	462 NDMM	MRD negativity rate	NR	Recruiting
NCT04194931	1	Single arm: BCMA-CART cells and CD19-CART cells	20 R/R MM	ORR, MRD negativity rate, PFS, OS	NR	Recruiting
NCT04191616 (SELECT)	2	Single arm: KPd	85 R/R MM to R and previously exposed to Dara	MRD negativity rate	10^−5^/NGS	Not yet recruiting
NCT04133636	2	Single arm: JNJ-68284528 (BCMA CAR-T therapy)	80 R/R MM	MRD negativity rate	10^−5^/NR	Recruiting
NCT04124497	2	Single arm: DaraPd	45 R/R MM with del(17p) and not previously treated with Dara	MRD negativity rate	10^−5^ /NGS (ClonoSEQ assay)	Active, not recruiting
NCT04091126	3	Belantamab Mafodotin+VRd vs. VRd alone	810 NDMM non-eligible for ASCT	DLTs, SAEs, MRD negativity rate, PFS	10^−5^ /NGS	Recruiting
NCT03948035	3	Elo-KRd vs. KRd prior to and after ASCT and maintenance with Elo-R vs. R alone	576 NDMM eligible for ASCT	MRD negativity rate, PFS	NR/MFC	Recruiting
NCT03896737	2	DaraVCd vs. VTd prior to and after ASCT and maintenance with Dara-Ixa vs. Ixa alone	400 NDMM	PFS, MRD negativity rate	10^−5^ /NGS (ClonoSEQ assay)	Recruiting
NCT03815279 (iStopMM)	2	Intermediate-risk sMM: Rd High-risk sMM & MM: KRd	80 sMM and MM	MRD negativity rate	NR/NGS	Enrolling by invitation
NCT03652064	3	DaraVRd followed by DaraRd vs. VRd followed by Rd	395 NDMM for whom ASCT is not planned as initial therapy	MRD negativity rate	10^−5^ /NGS	Active not recruiting
NCT03617731	3	Isa-RVd vs. RVd for induction, ASCT and Isa-R vs. R for maintenance therapy	662 NDMM eligible for ASCT	MRD negativity rate after induction, PFS after 2nd randomization	10^−5^/NGF	Recruiting
NCT03500445	2	Single-arm: DaraKRd as initial therapy	75 NDMM eligible or not for ASCT	sCR rate, MRD negativity rate	NR/NGS	Recruiting
NCT03290950	2	Single-arm: DaraKRd	41 NDMM	MRD negativity rate	NR/NR	Recruiting
NCT02253316	2	Ixa-Rd as consolidation post ASCT + Ixa or R for maintenance	236 NDMM	MRD negativity rate at d112 post consolidation	NR/NGS (ClonoSEQ assay)	Active, not recruiting
NCT03104842	2	Isa-KRd as consolidation and Isa-KR as maintenance	153 NDMM eligible and not ASCT	MRD negativity rate	NR/NGF	Recruiting
NCT03477539	2	Single arm: Dara as consolidation, ASCT, DaraR for maintenance	50 MM patients eligible for ASCT with any prior induction therapy	MRD negativity rateat d100 post ASCT	NR/MFC	Recruiting

*Dara, Daratumumab; d, Dexamethazone; Elo, Elotuzumab; Isa, Isatuximab; Ixa, Ixazomib; K, Carfilzomib; P:Pomalidomide; R, Lenalidomide; V, Velcade; MM, Multiple Myeloma; sMM, Smoldering MM; NDMM, Newly-diagnosed MM; R/R, Relapsed/Refractory; CAR, Chimeric antigen receptor; BCMA, B-cell maturation antigen; ASCT, Autologous stem-cell transplantation; MRD, Minimal residual disease; NGS, Next-generation sequencing; NGF, Next-generation flow cytometry; MFC, Multicolor flow cytometry; DLT, dose- limiting toxicities; SAEs, Serious adverse events; sCR, Stringent complete response; ORR, Overall response rate; PFS, Progression free survival; OS, qurvival; NR, Not reported*.

## MRD in Therapeutic Decisions

Could MRD be the main driver in daily clinical practice and the major determinant for therapeutic decisions? For some hematological malignancies and especially leukemias (i.e., ALL, CLL, CML, APL), treatment decisions are often guided by the MRD result (101–103), while for others the clinical application of MRD is still doubtful (104, 105). Despite the consensus agreement of the prognostic impact of MRD in MM, the question is still open. At present, there is no established indication for advantageous therapeutic tailoring strategies based on the MRD result. However, the utility of MRD is offering a great potential to this direction and could be informative for several debatable aspects in clinical management (32, 106).

## Early, Delayed or No ASCT for Transplant Eligible Patients?

Results from the IFM/DFCI2009 study have shown that patients with upfront ASCT after 3 cycles of RVd induction experienced better therapeutic responses than those treated with 8 cycles RVd therapy and delayed ASCT (74). Nonetheless, patients who achieved MRD negativity <10^−6^ in both arms showed similar PFS and OS implying that early ASCT did not offer any additional benefit in cases who had already achieved deep MRD negative responses. Similar results came up in the recent study by Paiva et al. demonstrating that patients achieving undetectable MRD before or after HDT/ASCT had identical OS (66). Moreover, in the FORTE trial, where the arm of 4 induction KRd cycles followed by ASCT and 4 cycles of KRd concolidation (KRd-ASCT-KRd) was compared with the arm of 12 cycles of KRd (KRd12), initial data showed that the achievement of MRD negativity identified a subgroup of patients with R-ISS1 that had comparable outcomes in both arms (13). Early relapse occurred more often in the KRd12 arm, mainly due to the significantly lower rate of early relapses in R-ISS2 and R-ISS3 patients in the KRD-ASCT-KRD arm. Overall, these observations raise the question whether ASCT should be blindly applied to all transplant eligible-patients, or whether for patients who achieve deep MRD negative responses after induction therapy, stem cells should be harvested and kept for ASCT at a later stage after disease progression (32). While prospective clinical trials are needed to address this question, it has been sensibly suggested that in real world, for patients with limited access to novel agents or centers providing a reliable sensitive MRD evaluation, and for cases with aggressive clinical features, ASCT should still remain the standard of care (106, 107).

## Need for Consolidation After ASCT?

In the current clinical setting, consolidation therapy consisting of additional induction cycles, tandem ASCT or new therapeutic regimens is used to improve disease responses after ASCT with limited toxicity. The impact of consolidation therapy in improving VGPR and/or CR rates post-ASCT is relatively clear, though its apparent clinical impact on outcome remains controversial (71, 108–112). The clinical utility and the high depth of modern MRD assays may prove very beneficial to this point. We have recently reported that four cycles of KRd consolidation therapy in 39 patients who were MRD positive post-ASCT have significantly improved the depth of response (in 81% of cases) and changed the MRD status from positive to undetectable in 68% of them (113).

Future large randomized trials are needed to testify for consolidation superiority and if it is necessary in the clinical setting. An upcoming phase 2 single arm clinical trial (NCT04140162) will test whether the combination of DaraRd induction therapy followed by DaraVRd consolidation, if needed, will result in a higher rate of undetectable MRD compared to the standard of care. In this decision-making trial, containing both eligible and non-eligible patients for ASCT, consolidation will be applied only to those patients with MRD positivity after induction therapy. As MRD is about to be repeatedly evaluated, it is expected to show how many patients that were MRD positive after induction, turned to an MRD negative status due to DaraVRd consolidation.

In another ongoing phase 2 clinical trial (MASTER trial -NCT03224507-), patients are receiving Dara-KRd as induction therapy, followed by ASCT and 0, 4 or 8 cycles of Dara-KRd consolidation according to MRD status at each phase of therapy. In particular, MRD is evaluated at the end of induction, post-transplantation and during each 4-cycle block of Dara-KRd consolidation, and those patients who remain MRD <10^−5^ after two consecutive evaluations will discontinue therapy and will be monitored for MRD resurgence after 6 and 18 months. Patients with detectable MRD will complete all cycles of consolidation therapy and if MRD persists, patients will receive lenalidomide maintenance until disease progression or until patients show intolerance to treatment. The initial results of this study were recently released showing that MRD guided decision-making is feasible since MRD-based consolidation has increased the rates of deep responses, whereas on the other hand no patients relapsed or experienced resurgence of MRD after treatment discontinuation (114). Obviously, these promising results are still very preliminary with a short follow-up period and needs to be verified in the long run.

## What is the Optimal Duration of Maintenance Therapy?

The optimal duration of maintenance therapy is not well-demonstrated in the clinical management of MM. Current data rather support an improved impact of continuous therapy following ASCT, but cost-of-care and toxicity-implications due to long-term maintenance should be taken into consideration, especially in the context of a non-dramatic increase of definite impact to OS (32). More sensibly though, in the relapsed/refractory setting, where CR is difficult to achieve, a continuous approach of maintenance therapy to delay symptom initiation looks more profitable. However, there are still concerns regarding the decrease of the administered therapy and treatment fatigue that may be caused by frequent intravenous/subcutaneous-based combination therapies (101). Many ongoing clinical trials are designed to address this point with randomizations toward maintenance cessation upon achievement of sustained MRD negativity (Table 3) and are about to provide insight of the actual impact of indefinite treatment upon reaching deep responses. Accordingly, trials for MRD+ patients with randomization toward indefinite maintenance vs. switching therapy to a new class of drugs would be sensible (32).

**Table 3:** Ongoing clinical trials including MRD status in patients' enrollment and/or MRD-driven interventions.

**Identifier**	**Phase**	**Regimen/Purpose**	**Subjects**	**MRD-driven decision**	**Primary endpoint**	**Status**
NCT04108624 (MRD2STOP)	PO	Maintenance cessation	56 multimodality^£^MRD^neg^ MM patients on a single-agent maintenance for ≥1year	Maintenance cessation	MRD conversion rate, PFS, OS	Not yet recruiting
NCT04221178	PO	Maintenance cessation	50 MRD^neg^ MM patients for ≥3 years while on continuous maintenance	Maintenance cessation	MRD negativity rate (10^−5^) a year after enrolling	Recruiting
NCT03490344	2	Daratumumab effect on MRD^pos^ patients post induction	25 MRD^pos^ patients post induction with without consolidative HDT/ASCT	-	MRD negativity rate by MFC	Recruiting
NCT03992170 (DAR4MM)	2	Daratumumab effect on MRD^pos^ patients	50 MRD^pos^ patients with ≥VGPR after any previous therapy	All patients will receive Dara for 24 weeks MRD^neg^ (NGF): treatment cessation MRD^pos^: Daratumumab every 4 weeks for 80 more weeks	MRD negativity rate	Recruiting
NCT03901963 (AURIGA)	3	DaraR vs. R alone as maintenance treatment	214 MRD^pos^ (≥10^−5^)patients post ASCT	-	MRD conversion rate tested by NGS (10^−5^)	Recruiting
NCT03697655 (PREDATOR)	2	Preventive role of Daratumumab (Dara vs. no intervention) in reappearance of MRD	274 MRD^neg^ patients after one or two prior lines of therapy	-	EFS	Recruiting
NCT02389517	2	Ixa-Rd vs. R alone as maintenance therapy	86 MRD^pos^ patients after ASCT	-	MRD negativity rate by MFC	Recruiting
NCT02969837	2	Elo-KRd as initial therapy	55 NDMM non-transplant or transplant eligible agreed to defer ASCT	All with receive Elo-KRD for 12 cycles and then: MRD^neg^: Elo-Rd maintenance until PD MRD^pos^: Elo-KRd for 6 more cycles and then Elo-Rd maintenance until PD	sCR rate, MRD negativity rate by NGS (clonoSIGHT)	Recruiting
NCT04071457 (DRAMMATIC)	3	DARArHuPH20 + R vs. R alone as maintenance therapyto direct therapy duration	1100 patients post ASCT	After 2 years of maintenance with each arm:MRD^pos^ >10^−6^: Continue with assigned treatment MRD^neg^ (≤ 10^−6^): Randomization to either stop or continue assigned treatment for up to 7 years	OS	Recruiting
NCT02659293	3	KRd vs. R alone after ASCT	180 post ASCT that received a maximum of 2 induction regimens and have ≥SD at d100 post ASCT	Carfilzomib cycles 5–8 for MRD- patients that have no risk factors at the end of cycle 6Carfilzomib: cycles 5 - 36 for MRD^pos^ patients with high risk factors at the end of cycle 6	PFS	Recruiting
NCT04096066	3	KRd vs. Rd alone	340 elderly NDMM not eligible for ASCT	Patients with ≥VGPR & MRD^neg^ (10^−5^) for ≥ 1 year in the KRD arm will stop K (after ≥ 2 years of treatment) and continue with RD until PD or intolerance	MRD negativity rate, PFS	Recruiting
NCT04140162	2	DaraRd induction ± DaraVRd consolidation + DaraR maintenance	50 NDMM eligible and not for ASCT	Only those with MRD positive status after 6 cycles of induction will receive consolidation	MRD negativity rate after induction and/or consolidation	Not yet recruiting
NCT03710603 (PERSEUS)	3	DaraVRd arm: DaraVRd for induction and consolidation, DaraR for maintenance VRd arm: VRd for induction and consolidation, R for maintenance	690 NDMM eligible for ASCT	Patients in DaraVRd group with sustained MRD negativity (10^−5^) for 12 months and minimum 24 months of maintenance will stop Dara until PD or intolerance Upon recurrence of MRD or loss of CR, patients will restart Dara until PD or intolerance	PFS	Recruiting
NCT03224507 (MASTER)	2	DaraKRd for induction, ASCT ± DaraKRd consolidation ± R maintenance	82 NDMM eligible for ASCT	MRD (10^−5^) is evaluated post induction, post ASCT and during each 4-cycle block of Dara-KRd consolidation MRD^neg^ patients after two consecutive evaluations will stop therapy and will be monitored for MRD resurgence (In 6 and 18 months. MRD^pos^ patients post ASCT will complete all cycles of consolidation and if MRD persists, they will receive R maintenance until PD or intolerance	MRD negativity rate by NGS (clonoSEQ)	Recruiting

*Dara, Daratumumab; d, Dexamethazone; Elo, Elotuzumab; Ixa, Ixazomib; K, Carfilzomib; R, Lenalidomide; V, Velcade; MM, Multiple Myeloma; NDMM, Newly-diagnosed MM; ASCT, Autologous stem-cell transplantation; MRD, Minimal residual disease; NGS, Next-generation sequencing; NGF, Next-generation flow cytometry; MFC, Multicolor flow cytometry; sCR, Stringent complete response; EFS, Event-free survival; PFS, Progression free survival; OS, Overall survival; PO, Prospective observational*.

£ *Multimodality MRD negativity, MRD negativity by PET/CT and flow cytometry or NGS*.

Similar questions could be also made for the non-transplant eligible group of patients where the advantageous impact of continuous treatment on OS is not well demonstrated. Again, MRD-directed clinical trials where MRD negative patients would be randomized to either stop therapy or continue until disease progression or intolerance would prove informative.

## Defining Treatment Failure and MRD-Driven Treatment Intervention

At present, we cannot recommend MRD as the absolute goal of treatment, though current evidence supports its promising role as a reliable representative to standard clinical endpoints that could provide efficacy evaluation of an administered treatment in a much more expeditious fashion. The design of ongoing clinical trials is allowing for testing this hypothesis; if the results of such trials prove successful, that will emerge MRD negativity as the optimal endpoint for efficient responses to treatment and probably to a redefinition of treatment failure, paving the way for MRD-based adapted approaches.

The current treatment paradigm in MM is to continue treatment until progression, and, for relapsing patients, a relative delay of retreatment initiation until redevelopment of CRAB features. As mentioned, there are now several ongoing trials to address the potential of stopping or decreasing administered therapy upon achievement of MRD negativity (Table 3), however there are not yet designed trials to explore the potential benefit of an early treatment intervention based only on the reversal of the MRD status from negative to positive. Such trials would need to start from a common basis of MRD negative background after a particular therapy, with frequent subsequent MRD assessments, and upon reappearance of MRD, patients should be randomized to either receive an early salvation therapy or wait for it until the appearance of standard disease progression events. In an alternative approach, MRD positive “relapsed” patients could be further randomized on the basis of their tumor burden levels, to those who start immediately salvation therapy due to their high tumor burden and to those who have a more delayed initiation by the time that MRD reaches a predefined value. Furthermore, MRD assessment could better characterize the group of patients with symptomatic MM and MGUS-like biological behavior. It would be valuable to identify these non-responding, non-progressing patients at diagnosis and, possibly, avoid over-treatment in those with no end-organ damage.

## Conclusions

Modern therapeutic options and extensive improvements in the management of MM have remarkably improved the efficacy of administered treatments and consequently prolonged progression free periods and patients' survival. The emergence of MRD has proven as the most reliable marker of response and subsequent prognostication. Based on current evidence, the achievement of MRD negativity can overpass other prognostic factors, reverse previous risk stratification and delay a future relapse, irrespective of the assigned therapeutic regimen. It is therefore rational that many ongoing clinical trials are now considering MRD negativity as an exclusive or additional primary endpoint. Moving a step further, the role of MRD status in defining treatment decisions seems feasible and relevant designed trials will soon evaluate the effectiveness of tailoring MRD-strategies giving insights on how MRD could become an invaluable tool in daily clinical practice.

In parallel however, there are still several open questions of MRD assessment and considerations of further improvements for a better utilization of the MRD result in the clinical management. The extensive research together with the results from several ongoing trials as well as the access to novel and more sensitive methodologies for an ever deeper response assessment, form a challenging and rapid changing field of MRD in the clinical setting. Nonetheless, based on the myriad new opportunities arisen, MRD is anticipated to have the pivotal role in modern therapeutic strategies driven by the individualized patient's MRD-based response profile.

## Author Contributions

IK and IN-S performed the literature review and drafted the manuscript. All authors revised critically the manuscript and gave their final approval for publication.

## Conflict of Interest

The authors declare that the research was conducted in the absence of any commercial or financial relationships that could be construed as a potential conflict of interest. The reviewer MB declared a past co-authorship with one of the authors ET.

## Abbreviations

AF: Allele fractions
ASCT: Autologous stem cell transplantation
ASO PCR: Allele specific oligonucleotide PCR
BM: Bone marrow
CDR: Complementarity determining region
cfDNA: Cell-free DNA
CTCs: Circulating tumor cells
ctDNA: Circulating tumor DNA
CR: Complete Remission
CT: Computed tomography
DCE: dynamic contrast-enhanced
DWI: diffusion-weighted imaging
EMD: Extramedullary disease
FDG-PET: ^18^fluoro-2-deoxyglucose positron emission tomography
IMWG: International myeloma working group
LOD: Limit of detection
MFC: Multicolor flow cytometry
miRAMM: Monoclonal immunoglobulin rapid accurate molecular mass
MM: Multiple Myeloma
MRD: Minimal residual disease
MRI: Magnetic resonance imaging
NGF: Next-generation flow cytometry
NGS: Next-generation sequencing
OS: Overall survival
PB: Peripheral blood
PFS: Progression free survival
PR: Partial response
(R)-ISS: (Revised) International staging system
RQ-PCR: Real-time quantitative PCR
SUVmax: Maximum standardized uptake value
VGPR: Very good partial response.
